# Evaluation of Antimalarial Activity and Toxicity of a New Primaquine Prodrug

**DOI:** 10.1371/journal.pone.0105217

**Published:** 2014-08-18

**Authors:** Marcelo Gomes Davanço, Anna Caroline Campos Aguiar, Leandro Alves dos Santos, Elias Carvalho Padilha, Michel Leandro Campos, Cleverton Roberto de Andrade, Luiz Marcos da Fonseca, Jean Leandro dos Santos, Chung Man Chin, Antoniana Ursine Krettli, Rosangela Gonçalves Peccinini

**Affiliations:** 1 Departamento de Princípios Ativos Naturais e Toxicologia, Faculdade de Ciências Farmacêuticas, Universidade Estadual Paulista – UNESP, Araraquara, São Paulo, Brazil; 2 Centro de Pesquisas René Rachou, FIOCRUZ, Belo Horizonte, Minas Gerais, Brazil; 3 Departamento de Fisiologia e Patologia, Faculdade de Odontologia de Araraquara, Universidade Estadual Paulista – UNESP, Araraquara, São Paulo, Brazil; 4 Departamento de Análises Clínicas, Faculdade de Ciências Farmacêuticas, Universidade Estadual Paulista – UNESP, Araraquara, São Paulo, Brazil; 5 Laboratório de Pesquisa e Desenvolvimento de Fármacos – Lapdesf, Faculdade de Ciências Farmacêuticas, Universidade Estadual Paulista - UNESP, Araraquara, São Paulo, Brazil; Centro de Pesquisa Rene Rachou/Fundação Oswaldo Cruz (Fiocruz-Minas), Brazil

## Abstract

*Plasmodium vivax* is the most prevalent of the five species causing malaria in humans. The current available treatment for *P. vivax* malaria is limited and unsatisfactory due to at least two drawbacks: the undesirable side effects of primaquine (PQ) and drug resistance to chloroquine. Phenylalanine-alanine-PQ (Phe-Ala-PQ) is a PQ prodrug with a more favorable pharmacokinetic profile compared to PQ. The toxicity of this prodrug was evaluated in *in vitro* assays using a human hepatoma cell line (HepG2), a monkey kidney cell line (BGM), and human red blood cells deficient in the enzyme glucose-6-phosphate-dehydrogenase (G6PD). In addition, *in vivo* toxicity assays were performed with rats that received multiple doses of Phe-Ala-PQ to evaluate biochemical, hematological, and histopathological parameters. The activity was assessed by the inhibition of the sporogonic cycle using a chicken malaria parasite. Phe-Ala-PQ blocked malaria transmission in Aedes mosquitoes. When compared with PQ, it was less cytotoxic to BGM and HepG2 cells and caused less hemolysis of G6PD-deficient red blood cells at similar concentrations. The prodrug caused less alteration in the biochemical parameters than did PQ. Histopathological analysis of the liver and kidney did show differences between the control and Phe-Ala-PQ-treated groups, but they were not statistically significant. Taken together, the results highlight the prodrug as a novel lead compound candidate for the treatment of *P. vivax* malaria and as a blocker of malaria transmission.

## Introduction

Malaria is a major human infectious disease that affects 500 million individuals each year [1]. Although global malaria morbidity and mortality have decreased substantially, this disease still kills roughly 2000 people per day, most of whom are children in Africa [2]. Among the five parasite species that affect humans, *Plasmodium falciparum* is responsible for the most severe morbidities and worldwide number of deaths. Nowadays, the severity of malaria is aggravated by resistant *Plasmodium* strains, spread of insecticide-resistant vectors, and lack of vaccines and effective drugs [3].

The current drugs available are limited and unsatisfactory due to undesirable side effects, high cost and drug resistance (MDR) to antimalarials; in addition, most such blood schizonticides are not able to act against other parasite stages [4], and the combination of two or more drugs is used to reduce the risk of treatment failure [5].

Primaquine (PQ) is the only antimalarial active against gametocytes from all species of parasite, including chloroquine-resistant *P. falciparum* and latent liver forms responsible for relapsing malaria caused by *P. ovale* and *P. vivax*. Thus, PQ is used to interrupt disease transmission from the host to mosquitoes, and it has demonstrated blood schizontocidal activity [6]. The exact mechanism how PQ eliminates the parasite hypnozoites and gametocytes is still unknown, but its proposed mode of action includes a) impairment of the parasite mitochondria interfering with the ubiquinone function in the respiratory chain and b) high production of intracellular reactive species increasing oxidative stress [7], [8].

Despite these beneficial effects, PQ can cause blood toxicity such as methemoglobinemia, especially in patients with G6PD deficiency, resulting in severe hemolytic anemia [9]. Another limiting factor for PQ is its short half-life with fast deamination of PQ to its inactive metabolite carboxyprimaquine after drug administration in mammals [10].

A molecular modification strategy such as a prodrug approach has been used to improve the pharmacokinetic (PK) and pharmacodynamic (PD) properties of *O*-alkyl and *O*-aryl carbamate derivatives of PQ, which were synthesized to prevent oxidative deamination to carboxyprimaquine. *O*-Alkyl derivatives demonstrate high stability against chemical hydrolysis. Some derivatives such as ethyl and n-hexyl carbamates, given to a gametocytemic host, have been shown to reduce the number of infected mosquitoes, thereby being useful for blocking malaria transmission [11]. PQ dipeptides containing an imidazolidin-4-one moiety at the *N*-terminus have been synthesized and evaluated against malaria. The Ala-Ala-PQ derivative is more effective than PQ in reducing the transmission of the infection to mosquitoes by decreasing the number of oocysts in the midgut of the mosquitoes [12].

The use of peptides as carriers has been employed to obtain PQ-derivatives that are less toxic than unmodified PQ [13], [14], [15]. It is expected that the peptide derivatives exhibit increased water solubility and thus lower log P when compared with PQ. The increased water solubility generally brings favorable consequences to the PK profile and to the toxicological characteristics [16].

Chung and collaborators described the synthesis of the dipeptide phenylalanine-alanine-primaquine (Phe-Ala-PQ) as a prodrug of PQ. Despite the excellent anti-*Trypanosoma cruzi* activity of Phe-Ala-PQ, this dipeptide has not been evaluated against malaria. PK and hematotoxicity studies have been performed with this prodrug. The bioconversion of Phe-Ala-PQ to PQ has been observed *in vitro* and *in vivo*, where the PK parameters are more favorable with the prodrug due to low plasma fluctuations of PQ. The mean residence time (MRT) of PQ from prodrug is higher compared to PQ, showing that the prodrug approach could overcome the PK problems of PQ, such as short plasma half-life (ca. 6 h) [17], [18]. In addition, a hemolytic effect has been observed after the administration of multiple oral doses of PQ, whereas this hemolytic effect is not observed after Phe-Ala-PQ administration [17]. Therefore, in a continuing effort to develop new candidate drugs to treat malaria, we report here on the antimalarial activity, cytotoxicity, and biochemical, hematological and histopathological effects of the prodrug Phe-Ala-PQ, designed as an antimalarial agent.

## Materials and Methods

### Preparation and partition coefficient determination of Phe-Ala-PQ prodrug

The prodrug Phe-Ala-PQ was prepared by condensation between PQ, alanine and phenylalanine using coupling reactions in good yields (ca. 50%) according to procedures previously described in the literature [19]. In this study, all compounds were also analyzed by high performance liquid-chromatography (HPLC) and their purity was found to be over 98.5%.

The Phe-Ala-PQ partition coefficient (log P) was determined by an HPLC method according to OECD guidelines for testing chemicals [20]. A Waters Alliance HPLC system equipped with a UV-Vis 2487 detector was employed. The chromatographic analysis was performed on a Symmetry C_18_ HPLC column (4.6 mm×250 mm, 5 µm particle size) and the mobile phase was methanol:water (70∶30), with detection at 256 nm. A flow rate of 1.0 mL/min was used and 50-µL samples were injected into the chromatographic system. The run time was approximately 15 min. The analytical curve was constructed on the basis of the retention time of the standards (purchased from Sigma Aldrich, NJ, USA) butanone, acetanilide, phenol, phenacetin, benzene, toluene, chlorobenzene and thymol, dissolved in mobile phase at a concentration of 50 µg/mL. The substances were analyzed in triplicate and the average retention time for each standard was used. The capacity factor (k) was obtained by the equation [20]: k =  (t_R_ - t_0_) /t_0_, where t_R_ is the retention time of the test substance and t_0_ the dead-time. The log k values were correlated with the partition coefficients of the standards for construction of the analytical curve. The log P of Phe-Ala-PQ was calculated by the equation of the analytical curve. The coefficient of determination (r^2^) was also calculated to evaluate the linearity of the analytical curve.

### Animals

The biochemical, hematological and histopathological analyses were carried out in male Wistar rats (weighing 200–250 g). They were housed in wire cages, five animals per cage, with free access to food and water. The study protocol was approved by The Research Ethics Committee of the School of Pharmaceutical Sciences, UNESP, Araraquara (process 06/2009). The rats were divided into three groups and treated as follows: control group: saline by gavage (four times a day for 4 days) (n = 10); PQ diphosphate group: PQ diphosphate by gavage (2.44 mg/kg free base; four times a day for 4 days) (n = 10); and Phe-Ala-PQ group: prodrug by gavage (9.00 mg/kg; four times a day for 4 days) (n = 10). This dose and frequency were derived on the basis of the dose regimen of malaria treatment in humans by allometric scaling, and considering oral bioavailability (approximately 50%) of prodrug determined in a previous study [17]. After treatment with the drugs, the animals were decapitated for collection of blood and samples of liver and kidneys (preserved in 10% formaldehyde).

### Inhibition of *P. gallinaceum* sporogony in *Aedes fluviatilis* mosquitoes

The use of laboratory animals was approved by the Ethics Committee for Animal Use of the Oswaldo Cruz Foundation - Fiocruz (CEUA LW-23/13).

Phe-Ala-PQ was tested for its ability to inhibit the sporogonic development of *P. gallinaceum* in mosquitoes fed on infected-treated chickens, in experiments performed as described before [21] and briefly as follows. One-week-old chicks (*Gallus gallus domesticus*) previously inoculated with 10^6^ RBC infected with *P. gallinaceum,* had their parasitemia monitoried daily, by examining Giemsa-stained thin blood smears; they were used to feed the mosquitoes when reaching 2.8 – 3.8%. The *A. fluviatilis* mosquitoes were kept for 24 h without sugar in their diet, to improve their ability to take the blood meal from chickens. For each compound the same infected chicken was offered twice to feed clean mosquitoes (30 female mosquitoes per group), before treatment, (group 0 h). Drug treatment was immediately performed by gavage and the treated chicken was offered again to blood-feed another group of mosquitoes (test group) 4 h after treatment of the chicken with the drug. Phe-Ala-PQ was tested at 10, 5 and 1 mg/kg to provide doses of PQ at 1.9, 0.94 and 0.19 mg/kg, respectively, whereas PQ, a reference antimalarial, was used at 1.0 mg/kg, because of its toxicity. In each experiment, another non-treated *P. gallinaceum*-infected chick was used twice (time 0 h and 4 h later) to feed clean mosquitoes, serving as an internal control.

Seven days after the blood meal, the mosquitoes were dissected and their midgut was removed under a stereomicroscope (100x), stained with 0.5% mercurochrome, and examined under a light microscope (40x) for the oocyst count. The inhibition of sporogony was based on the number of oocysts found in the control mosquitoes (0 h), considered as 100% infection, and those found in the mosquitoes fed 4 h after drug-treatment. Two criteria were used to evaluate drug activity: the total number of infected mosquitoes and the mean number of oocysts, based on the examination of 20 mosquitoes in each group.

### *In vitro* cytotoxic effect of Phe-Ala-PQ

The cytotoxicity of Phe-Ala-PQ was assessed using a human hepatoma cell line (HepG2) and a kidney monkey cell (BGM), cultured in 75-cm^2^ sterile flasks in RPMI 1640 supplemented with 10% heat-inactivated fetal bovine serum and 40 mg/L gentamicin, in a 5% CO_2_ atmosphere at 37°C. The HepG2 (ATCC HB-8065) was originally received from the New University of Lisbon, Portugal, and BGM received as a gift from the Federal University of Minas Gerais and originally purchased from Rio de Janeiro Cell Bank (BCRJ 0049). When confluent, the cells were washed with RPMI culture medium, then trypsinized, distributed in a flat-bottom 96-well plate (5×10^3^ cells/well), and incubated for 18 h at 37°C for cell adherence as described elsewhere [22]. The compound (20 µL) was added to each well at various concentrations (1-1000 µg/mL) and the plate incubated for 24 h in a 5% CO_2_ atmosphere at 37°C. MTT [3-(4,5-dimethylthiazol-2-yl)-2,5-diphenyltetrazolium bromide] (5 mg/mL; 20 µL/well) was added to evaluate mitochondrial viability, and the plates were incubated again for 3 h, under the same culture conditions. The supernatant was carefully removed, and 100 µL of DMSO were added with mixing to dissolve the formazan crystals formed. The optical density was determined at 570 and 630 nm (background) (SpectraMax 340PC 384, Molecular Devices). Cell viability was expressed as the percentage of the control absorbance of the untreated cells after subtracting the appropriate background. The minimum lethal dose for 50% of the cells (MLD_50_) was determined as previously described [23].

### *In vitro* hemolysis assay

The test and control compounds (15–1000 µg/mL) were prepared in 0.2% (v/v) DMSO and incubated, at 37°C for 2 and 24 h in a shaking water bath, with a suspension of human erythrocytes (RBC, 2% hematocrit) obtained from either normal or glucose-6-phosphate dehydrogenase (G6PD)-deficient donors. The mixtures were centrifuged at 1000 g for 10 min, and the absorbance of the supernatants was measured at 540 nm in an ELISA plate reader (SpectraMax 340PC 384, Molecular Devices). The hemolytic rate was calculated using 0.05% saponin as the positive control for hemolysis of human RBC, which was considered 100% [24], [25]. The diagnosis of G6PD deficiency was performed using a technique based on the principle that hemoglobin oxidized to methemoglobin, by the action of sodium nitrite, is catalyzed by an enzyme, in the presence of methylene blue [26]. A final brown color indicates that a sample is positive for G6PD deficiency, while a bright red color is indicative of a normal RBC sample. We used RBC samples of four patients with G6PD deficiency, diagnosed by one of us (THK) [27]. The use of human RBC was approved by the Ethics Committee of CEPEM, (CAAE- 007 59912.0.0000.0011; October 9, 2012). All participants signed a written informed consent before blood collection. The consent was approved by the Ethics Committee of CEPEM.

### Biochemical and hematological evaluation

In biochemical analysis, aspartate aminotransferase (AST), alanine aminotransferase (ALT), gamma-glutamyltransferase (GGT), bilirubin, creatinine and urea kits were purchased from LabTest Diagnostica SA (Lagoa Santa,Minas Gerais, Brazil). Alkaline phosphatase (ALP) was assayed by the conversion of p-nitrophenyl phosphate to p-nitrophenol in a kinetic reaction, measured with a spectrophotometer at 405 nm. To determine the level of methemoglobin in relation to oxyhemoglobin, we used the method described by Naoum and collaborators [28]. The blood was hemolyzed and the hemoglobin stabilized in 60 mM phosphate buffer, pH 6.8. The levels of methemoglobin and oxyhemoglobin were determined by spectrophotometric absorption at 630 and 540 nm. The total and differential white blood cell (WBC) counts were performed using a Neubauer chamber and blood smears, respectively. The hematocrit was determined using conventional method by microcapillary centrifugation.

### Histopathological evaluation

The liver and kidney were removed from each rat to perform the histopathological evaluation. Tissue pieces were fixed in 10% formaldehyde and embedded in paraffin. Each organ was then cut into 4-µm thick sections and subjected to standard hematoxylin/eosin staining. Hepatic glycogen staining in sections was carried out using the standard periodic acid-Schiff (PAS) reaction [29], followed by hematoxylin counterstaining, and the periportal and centrilobular regions of the liver were analyzed according to Thoolen et al. [30]. Five periportal regions and five centrilobular regions were photographed in each animal, for a total of 300 photographs, and these images were adjusted using Pixelmator software (2.2.1). Nuclear hepatocyte glycogen levels were classified as: A- PAS<50% of cells, B- PAS≥50% of cells and C- PAS-negative. Additionally, liver sections were examined for the occurrence of microvesicular fatty changes, as well as the kidney sections for inflammatory infiltration, reduction of Bowman's spaces and changes in renal tubules. Statistical analysis was performed in Graphpad Prism 5.0b. Data for each organ analysis were expressed qualitatively and quantitatively, and normal distribution was verified (D'Agostin and Person). t-tests and chi-square tests were performed, and non-parametric data were evaluated using the Mann-Whitney or Kruskal-Wallis test for pair wise comparisons of groups. The significance level was set at 5%.

## Results

### Determination of Phe-Ala-PQ partition coefficient

The lipophilicity of PQ and Phe-Ala-PQ, expressed as log P values, was determined using the HPLC method [20]. Experimental data of log P for PQ and prodrug showed values of 2.1 and 0.94, respectively. The introduction of the dipeptide Phe-Ala in the PQ structure decreased log P and increased water solubility.

### Antimalarial activity

The mean numbers of oocysts in the group of mosquitoes fed on parasitized chicks before and after treatment are shown in Table 1. PQ caused a decrease from 60 (0 h) to 2.1 (4 h), representing 96% reduction in oocyst number. PQ from prodrug at 0.94 mg/kg and 1.9 mg/kg decreased the number of oocysts by 70% and 100%, respectively (Table 1).

**Table 1:** Effect of Phe-Ala-PQ and PQ on sporogonic cycle of *Aedes fluviatilis* mosquitoes fed on chickens infected with *P. gallinaceum* and treated orally with a single dose of the drug, before and four hours after treatment with the drugs.

Chicken treatment	Dose (mg/kg)	Mean oocyts ±SD/ in times	Mean oocyts ±SD/ in times	Reduction in oocyst number, %	Number of infected mosquitoes/total analyzed (%)	Number of infected mosquitoes/total analyzed (%)	Sporogony inhibition, %
		0 h	4 h		0 h	4 h	
**Phe-Ala-PQ (PQ from Phe-Ala-PQ)**	1.0 (0.19)	11±16	26±36	0	8/20(40)	13/20(65)	0
	5.0 (0.94)	19±23	6±6*	70	15/20(75)	13/20(65)*	10
	10 (1.9)	65±33	0±0*	100	18/20(90)	0/20(0)*	100
**PQ diphosphate**	1.0	60±84	2.1±4.3*	96	12/20(60)	6/20(30)*	50

*Statistical differences as compared to time 0 h (p<0.05), by non-parametric Mann–Whitney test.

The inhibition of sporogony was also observed for PQ from prodrug at 0.94 and 1.9 mg/kg. At these doses, the percentages of inhibition were 10 and 100%, respectively, while PQ diphosphate at 1.0 mg/kg produced 50% inhibition (Table 1). The results showed that PQ from prodrug at 1.9 mg/kg decreased the number of oocysts by 100%. These inhibition values were greater compared to PQ, suggesting that the prodrug can be used as an antimalarial and also for prophylaxis.

### Cytotoxicity study

The MDL_50_ values for PQ were 263 and 180 µg/mL for BMG (BCRJ 0049) and HepG2 (ATCC HB-8065) cells, respectively. Phe-Ala-PQ showed MDL_50_ values of 580 and 920 µg/mL (110 and 175 µg/mL PQ equivalents) for BMG and HepG2 cells, respectively. These results seen in table 2, demonstrated that compared to PQ, the prodrug was about two times less toxic to BGM cells and five times less toxic to HepG2.

**Table 2:** Cytotoxicity of Phe-Ala-PQ and PQ against a human hepatoma cell line (HepG2) and a monkey kidney cell line (BGM) determined by the MTT assay.

Drug	MDL_50_ µg/mL (Mean±SD)	MDL_50_ µg/mL (Mean±SD)
	BGM	HepG2
**Phe-Ala-PQ (PQ equivalents)**	580±86 (110)	920±1 (175)
**PQ**	263±4	180±24

Data expressed as the 50% minimal lethal dose (MDL50).

### Hemolysis in red blood cell G6PD deficient

At 1000 µg/mL (190 µg/mL PQ equivalents in prodrug), both compounds were hemolytic, but at 500 µg/mL (95 µg/mL PQ equivalents in prodrug), the percentage of hemolysis induced by PQ was 87.8% while the prodrug caused 51%. Phe-Ala-PQ did not cause hemolysis at concentrations equal to or less than 250 µg/mL (47.5 µg/mL PQ equivalents in prodrug) (Table 3).

**Table 3:** Percentage of *in vitro* hemolysis by Phe-Ala-PQ and PQ in G6PD-deficient human red blood cells.

Dose (µg/mL)	Hemolysis %	Hemolysis %
	Phe-Ala-PQ*	PQ
1000	100	100
500	51	87.8
250	0	50
125	0	0
62	0	0
31	0	0

*PQ equivalents: 190, 95, 47.5, 23.7, 11.8 and 5.9 µg/mL.

### Biochemical and hematological evaluation

Phe-Ala-PQ increased AST and ALT levels compared to the PQ and control groups (Table 4). However, the prodrug induced a reduction in ALP and GGT levels compared to PQ and control groups. The levels of total bilirubin of the control, PQ and Phe-Ala-PQ groups were 0.551, 0.797 and 0.711 mg/dL, respectively, and the direct bilirubin levels were 0.065, 0.228 and 0.147 mg/dL, respectively, for each group. PQ treatment raised creatinine levels (0.986 mg/dL), while the prodrug did not. In addition, urea levels were lower in the prodrug group (5.734 mg/dL) compared to the PQ (18.51 mg/dL) and control (30.29 mg/dL) groups.

**Table 4:** Biochemical analysis of serum of rats treated with multiple doses of Phe-Ala-PQ or PQ (mean ±CI 95).

	Control	PQ	Phe-Ala-PQ
**AST (U/L)**	198.3	64.99^a^	290.6^a,^ b
	(170.4–226.3)	(41.73–88.25)	(237.9–343.3)
**ALT (U/L)**	50.25	91.81^a^	185.7 ^a,^ b
	(46.45–54.05)	(73.91–109.7)	(171.2–200.4)
**ALP (U/L)**	109.5	125.6	93.52
	(94.09–124.9)	(78.77–171.7)	(81.42–105.6)
**GGT (U/L)**	39.11	22.88^a^	13.03^a,^ b
	(28.01–50.19)	(14.91–30.85)	(8.365–17.70)
**Total bilirubin (mg/dL)**	0.551	0.797^a^	0.711
	(0.491–0.609)	(0.563–1.031)	(0.418–1.001)
**Direct bilirubin (mg/dL)**	0.065	0.228	0.147
	(−0.006–0.296)	(0.106–0.343)	(0.031–0.262)
**Creatinine (mg/dL)**	0.428	0.986^a^	0.529
	(0.345–0.512)	(0.835–1.138)	(−0.382–2.591)
**Urea (mg/dL)**	30.29	18.51	5.734^a,^ b
	(25.54–34.89)	(8.505–28.52)	(3.351–8.118)

a : p<0.05 compared with control group;

b : p<0.05 compared with PQ group.

In the hematological analysis, the WBC count (total leukocytes, neutrophils, eosinophils, lymphocytes and monocytes), hematocrit, hemoglobin concentration and methemoglobin percentage were determined (Table 5). PQ and Phe-Ala-PQ increased lymphocytes and decreased neutrophils compared to the control group. PQ decreased monocytes when compared with control and Phe-Ala-PQ. There was no significant difference in hematocrit, hemoglobin concentration and methemoglobin percentage between the groups.

**Table 5:** Hemogram of rats treated with multiple doses of Phe-Ala-PQ or PQ (n = 30, mean ±CI 95).

		Control		PQ		Phe-Ala-PQ	
		Relative (%)	Absolute	Relative (%)	Absolute	Relative (%)	Absolute
			(No. cells)		(No. cells)		(No. cells)
**Neutrophils**	**Segmented**	22.9 (16.8–28.9)	1041 (807–1276)	11.6^a^ (8.15–15.0)	516^a^ (403–629)	9.00^a^ (2.55–15.4)	895 (163–1626)
	**Band**	0.20 (0.00–.60)	10 (0–34)	0.11 (0.00–0.36)	6 (0–19)	0	0
**Eosinophils**		1.10 (0.47–1.72)	51 (20–83)	2.10 (0.77–3.42)	97 (43–151)	0.40 (0.00–1.51)	40 (0–152)
**Lymphocytes**		69.5 (64.6–74.4)	3349 (2628–4069)	83.9^a^ (78.9–88.9)	4123 (3200–5046)	83.0^a^ (75.8–90.2)	7691^a^^,^ b (5947–9976)
**Monocytes**		6.30 (4.68–7.92)	305 (197–412)	2.30^a^ (0.86–3.73)	96^a^ (52–140)	7.60^b^ (0.06–15.1)	812^b^ (0–1728)
**Leukocytes (/mm^3^)**		4765 (3980–5549)		4840 (3973–5706)		8700^a^^,^ b (5623–11777)	
**Hemoglobin (mg/dL)**		12.39 (11.63–13.16)		10.08 (8.57–11.59)		11.76 (11.38–12.14)	
**Hematocrit (%)**		45 (43–46)		46 (44–48)		47 (45–48)	
**Methemoglobin (%)**		4,86 (3.25–6.48)		3.89 (3.39–4.40)		3.65 (3.45–3.85)	

a : p<0.05 compared with control group;

b : p<0.05 compared with PQ group.

### Histopathological evaluation

The macroscopic analysis of rats did not identify significant differences between the control, PQ and Phe-Ala-PQ groups (results not shown).

In the kidney, the analysis of all groups demonstrated normal structures with no alteration in glomeruli and renal tubules. The medullary region did not show inflammatory infiltrate or hemorrhagic process.

**Figure 1:**
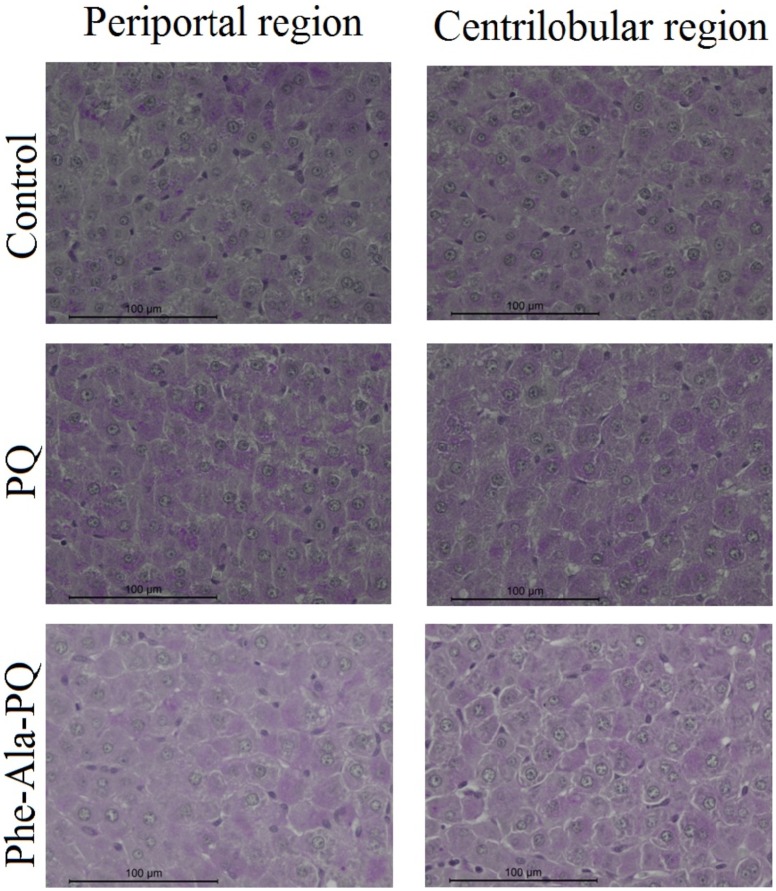
Photomicrographs of rat liver focusing on the periportal and centrilobular regions (hematoxylin/eosin staining, 40× original magnification, Bars  = 100 µm).

**Figure 2:**
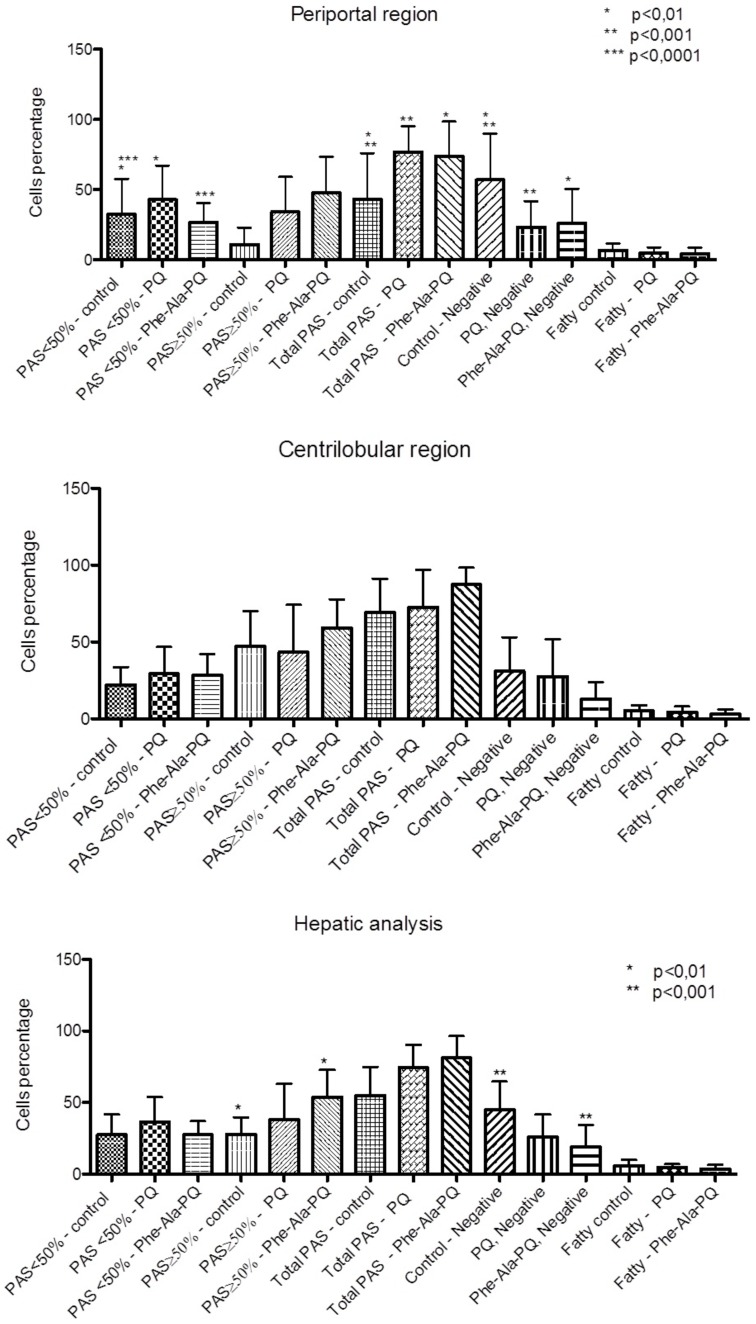
Hepatic analysis of glycogen: periportal region, centrilobular region and hepatic analysis (periportal + centrilobular regions).

The histopathological analysis of the liver demonstrated preserved normal structures in all groups (Figure 1). Liver glycogen analysis showed a distinct pattern in the different groups. In the periportal region, the PQ and Phe-Ala-PQ groups demonstrated more PAS-positive cells (total) compared to the control group. However, the finding of positive cells (<50%) demonstrated an increase in the Phe-Ala-PQ group and decrease in the PQ group. Analysis of the centrilobular region demonstrated no difference between the groups. The overall analysis of the liver demonstrated more PAS-positive cells (>50%) in the Phe-Ala-PQ group than control group (Figure 2).

## Discussion

The prodrug approach has been used to solve several PK, PD and stability problems of PQ. Despite adverse effects, PQ continues to be used in patients due its activity against latent liver forms and gametocytes. In addition, PQ is also active against relapsing malaria caused by *P. vivax* and *P. ovale* [8]. Interestingly, the antiparasitic activity of PQ is described not only against *Plasmodium spp.* but also for other infectious disease caused by *T. cruzi* [19], *Leishmania donovani* [31], [32], *Leishmania infantum* [33] and *Pneumocystis pneumonia* [34], [35], among others. This broad spectrum of action makes PQ an interesting prototype for molecular modification.

The use of PQ prodrugs containing amino acids and dipeptides seems to reduce toxicity and improve PK/PD parameters [11]–[16]. Phe-Ala-PQ, for example, is a prodrug containing the dipeptide phenylalanine-alanine as carrying group which shows *in vitro* and *in vivo* anti *T. cruzi* effects [17], [19]; however, the antimalarial effect has not yet been described. We have found that this compound causes less erythrocyte osmotic fragility and shows less fluctuation of plasma concentration [17]. The variation in PK parameters in Phe-Ala-PQ is due in part to increased water solubility caused by the introduction of the dipeptide. This modification allows sustained release of PQ by bioconversion of the prodrug, and it prolongs the mean residence time of PQ increasing its half life after oral administration [17]. All these effects are due to alterations in physico-chemical parameters such as partition coefficient (log P). In this work, we found experimental log P values for PQ equal to 2.1 compared to 0.94 for the prodrug. Log P is a physico-chemical property that not only indicated water-oil solubility but can also provide information about the ability of the compounds to cross cell membranes [16]. The next step was then to evaluate the *in vivo* and *in vitro* antimalarial activity of Phe-Ala-PQ and its ability to interfere in different stages of the parasite cycle.

To determine the antimalarial activity, one-week chicks were infected with *P. gallinaceum* via the intramuscular route. It was previously demonstrated that chickens with parasitemia below 6% provide reproducible results, so we used chicks with 2.8–3.8% parasitemia in this study [36]. PQ from Phe-Ala-PQ demonstrated activity against the sporogonic cycle, reducing the number of oocysts and sporozoites by 100% at 1.9 mg/kg. The model used here allows screening of drugs for the liver stage malaria parasite on the basis of the ability of such drugs to stop the development of gametocytes in the mosquito vector. In addition, the chicken malaria gametocyte screen has been shown to be more sensitive than the rodent screens in detecting useful compounds, with a minimum of false positive identifications [37]. Some *in vivo* experiments for screening antimalarial drugs include *P. cynomolgi*-induced malaria in Rhesus monkeys and *P. berghei*-induced malaria in a mouse model [38]. The *P. cynomolgi*-rhesus model has been used for decades to identify radical curative compounds [39], [40]. This model has been demonstrated to have observable hypnozoite forms and probably will be used in the next tests of antimalarial activity with this prodrug. Furthermore, *in vitro* screening assays have been recently investigated for the evaluation of novel hypnozoiticidal drugs because of a large numbers of new compounds. [41], [42].

After evaluation of antimalarial activity of Phe-Ala-PQ, we performed the *in vitro* and *in vivo* toxicological assays which included cytotoxicity, biochemical, hematological and histopathological evaluations. The low cytotoxicity of PQ derivatives has been described in the literature [43], but there are few data available. Kaur and collaborators reported that PQ derivatives were not cytotoxic using a mammalian kidney cell line (Vero) up to the highest concentration tested, 10 µg/mL [44]. In our study, we found that in a monkey kidney cell line (BGM) and human hepatoma cell line (HepG2) the prodrug was about two times less toxic than PQ to the BGM cells and five times less toxic to HepG2 cells at concentrations over 180 µg/mL.

Despite the low cytotoxicity of Phe-Ala-PQ, one of the serious and limiting problems of PQ derivatives is their hematological toxicity, mainly in patients with the glucose-6-phosphate dehydrogenase (G6PD) deficiency [45]. This effect is aggravated by the need of multiple administrations of high PQ doses to compensate for its low oral bioavailability [46]. We have previously reported that Phe-Ala-PQ induces less erythrocyte osmotic fragility than PQ [17] in non-G6PD-deficient RBC. This hemolytic effect seems to be related to better surfactant properties of PQ at high concentrations compared to the prodrug [47]. Here, we identified that hematological toxicity of Phe-Ala-PQ is also inferior to that of parent PQ in RBC deficient in G6PD. At 250 µg/mL Phe-Ala-PQ did not cause hemolysis while PQ induced 50% hemolysis at the same concentration. All these data are very promising and show that Phe-Ala-PQ is less cytotoxic and hematotoxic than the parent drug PQ.

Changes in biochemical parameters and WBC counts induced by PQ are controversial. Weerasinghe and co-workers found normal range values in patients treated with antimalarial drugs, including PQ, in the evaluation of WBC count, liver enzymes and creatinine levels [48]. On the other hand, Noel and collaborators reported that PQ is able to cause hepatic alterations at the transcriptional level after just a single dose, increasing hepatic stress markers in serum such as ALT and AST [49]. In the present work, we found that Phe-Ala-PQ increased AST and ALT levels compared to PQ, but in histopathological evaluation, the alterations were similar for the Phe-Ala-PQ and PQ groups.

Bilirubin is a degradation product of senescent erythrocytes and it is called unconjugated (indirect) before reaching the liver tissue. In the liver, bilirubin combines with endogenous substances to create a conjugated bilirubin (direct). Higher serum levels of direct or indirect bilirubin may indicate liver damage or increase in the rate of erythrocyte destruction [50]. In this work, no alteration in bilirrubin levels (total and direct) was observed in the Phe-Ala-PQ group.

Hematological analysis showed neutropenia and lymphocytosis in the PQ and Phe-Ala-PQ groups; and monocytopenia in the PQ rats. These alterations are related to the presence of PQ metabolites and multiple doses administered in animals [6], [48]. No significant changes were observed in methemoglobin percentage, hemoglobin concentration or hematocrit. Histopathological analysis of the kidney of the control, PQ and Phe-Ala-PQ groups demonstrated normal structures, with no alteration in the glomeruli and renal tubules. In the liver, the prodrug demonstrated fewer cells with fatty microvesicular changes. These biochemical and histological studies confirmed the lower toxicity of the prodrug compared to PQ.

## Conclusion

A novel dipeptide primaquine prodrug (Phe-Ala-PQ) was prepared and its antimalarial and toxicological profile was evaluated using *in vitro* and *in vivo* assays. The prodrug was found to be more soluble in water than the parent drug PQ. Evaluation of antimalarial activity demonstrated that PQ from the prodrug is effective against the sporogonic cycle, reducing the number of oocysts and sporozoites by 100% at 1.9 mg/kg. Phe-Ala-PQ was less cytotoxic than PQ in a monkey kidney cell line (BGM) and human hepatoma cell line (HepG2). At 250 µg/mL, Phe-Ala-PQ did not cause hemolysis in G6PD-deficient RBC, while PQ induced 50% hemolysis at the same concentration. Although the levels of ALT and AST were increased in the Phe-Ala-PQ group, histopathological analysis showed no changes compared to the PQ group. We did not observe a histological difference between the control and Phe-Ala-PQ groups with regard to the kidney. The liver of animals given the prodrug demonstrated a lower percentage of cells with fatty microvesicular changes compared to the PQ group. All the results herein presented point to the prodrug Phe-Ala-PQ as a novel lead drug candidate for new studies of antimalarial activity with other models.
